# Efficacy of isoproterenol in the evaluation of dormant conduction and arrhythmogenic foci identification in atrial fibrillation ablation

**DOI:** 10.1186/s12872-020-01685-w

**Published:** 2020-08-31

**Authors:** Yusuke Sakamoto, Hiroyuki Osanai, Shotaro Hiramatsu, Hikari Matsumoto, Kensuke Tagahara, Hirotaka Hosono, Shun Miyamoto, Hiroto Uno, Hideki Kurokawa, Shun Kondo, Kotaro Tokuda, Takahiro Kanbara, Yoshihito Nakashima, Hiroshi Asano, Masayoshi Ajioka

**Affiliations:** Department of Cardiology, Tosei General Hospital, 160 Nishi-Oiwake-cho, Seto-city, Aichi 489-8642 Japan

**Keywords:** Isoproterenol, Atrial fibrillation, Catheter ablation, Pulmonary vein isolation, Dormant conduction

## Abstract

**Background:**

Catheter ablation for atrial fibrillation (AF) is an established therapy. However, postoperative recurrence is a serious issue caused by the reconduction of the isolated pulmonary veins (PV) and the onset of non-PV foci. The objectives of this study were to elucidate dormant conduction, confirm PV arrhythmia substrate, induce non-PV foci after PV isolation, and assess the acute efficacy of high dose isoproterenol (ISP) when administered in addition to adenosine.

**Methods:**

The study consisted of 100 patients with drug-refractory AF (paroxysmal and persistent) who underwent ablation therapy (either radio-frequency or cryoballoon ablation) as the first-line of therapy at our hospital. All patients first underwent PV isolation (PVI) and were administered adenosine followed by ISP (6 μg × 5 min). The effects were observed, and the therapeutic strategy was evaluated.

**Results:**

Persistent dormant conduction due to ISP administration was observed in 13 patients. In over half of the patients, arrhythmia substrates were identified in the PV. Ten patients presented with persistent PV firing. The ablation of non-PV foci was additionally performed in 23 patients.

**Conclusions:**

We found that dormant conduction, as a result of ISP administration, is persistent and ISP is useful when performing an ablation. In addition, ISP administration is useful for the identification of PV arrhythmia substrates and induction of non-PV foci. However, the effectiveness of ISP may be partially due to the complementary effect of adenosine, and, therefore, a combination of the two drugs seems preferable.

## Background

A large body of evidence establishes catheter ablation as an effective treatment for atrial fibrillation (AF); and thus, it is currently the first-line treatment for AF [1–3]. However, postoperative recurrence is a serious problem caused by reconduction of the previously separated pulmonary veins (PV) and the onset of non-PV foci. There is evidence that ISP can be used to induce non-PV foci [4], and, while there are medical facilities that use it for this purpose, no studies have evaluated its use in dormant conduction and the identification of arrhythmia substrates. In this study, we anticipated to further improve the cure rate of atrial fibrillation ablation by assessing the effectiveness of ISP.

The objectives of this study were to elucidate dormant conduction after PV isolation (PVI), confirm PV arrhythmia substrate, induce non-PV foci, and observe the acute efficacy of high dose isoproterenol (ISP) loading in addition to adenosine.

## Methods

### Patient selection

Subjects consisted of 100 consecutive patients with drug-refractory AF (paroxysmal or persistent) who underwent ablation therapy (either radio-frequency or cryoballoon ablation) as the first-time therapy at Tosei General Hospital between June 2019 and January 2020.

This was a prospective single-center observational study. All patients provided written informed consent for the ablation procedure and enrollment in our ablation registry. This study was conducted after obtaining the approval of the hospital Institutional Review Board.

### Exclusion criteria

Patients with the following risk factors for ISP administration were excluded: hypertrophic cardiomyopathy, severe aortic stenosis, severe coronary artery disease, and an ISP allergy.

### Ablation protocol

Bearing in mind five-times the half-life of antiarrhythmic drugs, their administration was discontinued before the ablation procedure. Cases with left atrial thrombus were detected by measuring coagulation markers, esophageal echocardiography, or contrast CT scans and were excluded. The administration of all oral anticoagulants (warfarin and direct oral anticoagulants) was continued throughout the ablation procedure.

The ablation procedure was performed under sedation using propofol and dexmedetomidine. A bolus dose of heparin was administered after puncturing the inguinal and left subclavian vein to maintain the activated coagulation time (ACT) level during the ablation procedure at a minimum of 300 s. A 20-pole catheter was inserted into the coronary sinus (CS) via the left subclavian vein for cardiac defibrillation, and the superior vena cava (SVC), right atrial (RA), and CS potentials were also obtained. A trans-septal puncture was performed using an intracardiac catheter ultrasound. A 20-pole ring electrode catheter was then inserted via an SL0 sheath (Abbott) and either a THERMOCOOL SMARTTOUCH SF Catheter (Johnson & Johnson) or a TactiCath SE irrigation catheter (Abbott) was inserted via a steerable sheath (Agilis, Abbott) and installed in the left atrium (LA). Cardioversion was performed in patients with persistent atrial fibrillation immediately prior to ablation. Catheter ablation was performed using a 3D mapping system (CARTO3/Johnson & Johnson, or Ensite Velocity/Abbott).

**Fig. 1:**
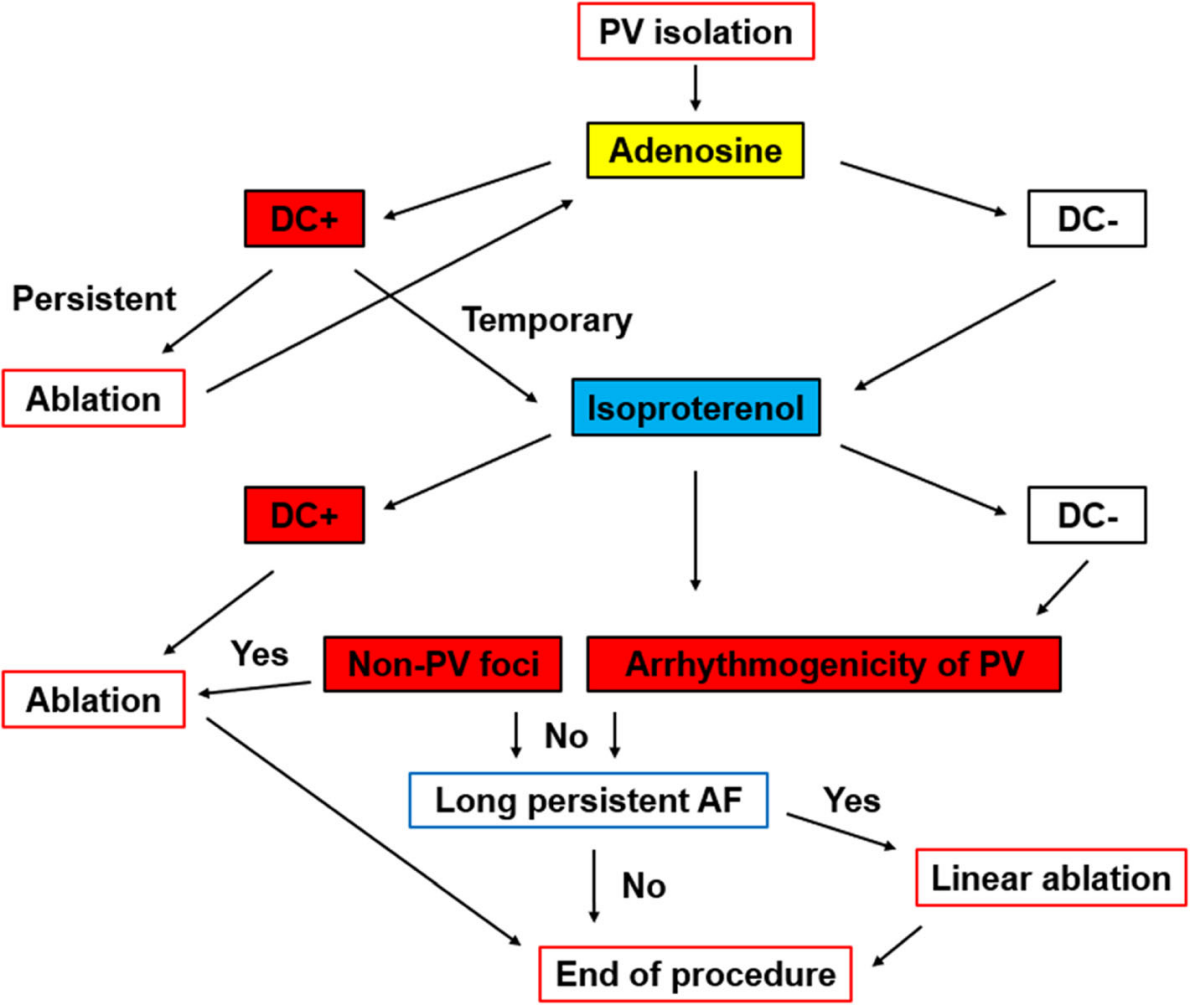
The ablation protocol utilized in the study. PV: pulmonary vein; DC: dormant conduction

The ablation therapy protocol is shown in Fig. 1. First, all patients underwent a PVI. A blockage in both directions confirmed the completion of the PVI process. In addition, the activity within the isolated PV was checked prior to drug administration.

Following PVI, adenosine was administered to reveal the sites of dormant conduction. Adenosine was not used in asthmatic patients. In patients who underwent a simultaneous separation of the PV, electrodes were attached to both sides of the PV and 30 mg of adenosine was administered, whereas, in those who underwent an individual separation or cryoablation, 30 mg of adenosine was injected into the superior and inferior PV. In cases where dormant conduction occurred continuously as a result of adenosine, additional ablation procedures were performed. The results of the additional ablation procedure were then confirmed by administering an additional 30 mg of adenosine. In cases where the administration of adenosine did not reveal the dormant conduction or in those in whom the dormant conduction was temporary, ISP was administered.

A high dose of ISP was administered at a rate of 6 μg/min for 5 min. This dose was determined based on our experience in several patients at the pre-study stage that revealed no worsening of circulatory dynamics at this dose. An additional electric current was applied to patients in whom dormant conduction was revealed following the administration of ISP.

In all cases, the additional ablation led to the formation of continuous ablation focal points in the region surrounding the site of re-conduction. The results obtained from our check of the PV arrhythmia substrates were categorized as no response, elevated automaticity, three consecutive firings, and continuous firing.

Non-PV foci are defined as premature atrial contractions associated with AF induction. They either induce atrial tachycardia or repeat frequently. In patients in whom non-PV foci were induced, we mapped the origin and performed an additional ablation. In some cases, in addition to the above-mentioned induction by drug administration, we also conducted investigations by performing high-frequency atrial pacing and, when necessary, defibrillation, in cases of atrial fibrillation.

After administering ISP, observations made during the 15 min duration of action helped to determine the following: in cases where an obvious PV arrhythmia substrate was confirmed and non-PV foci were not confirmed, the procedure was concluded after performing only PVI. However, in those with a long-lasting persistence of AF for at least 1 year and in whom neither PV arrhythmia substrate nor non-PV foci could be confirmed, left atrial linear ablation was additionally performed. The site where the line was created was determined by the operator taking into consideration the patient’s disease history.

### Statistical analyses

The continuous data were expressed as mean ± standard deviation for the normally distributed variables and as median [25th and 75th percentiles] for the non-normally distributed ones.

## Results

### Patient characteristics

The background characteristics of the 100 patients are shown in Table 1. Adenosine was not utilized in five of these patients with asthma. There were 61 men with a mean age of 69.3 ± 9.6 years, and 12 of the 32 patients with persistent AF had the disease for at least 1 year. The mean duration of AF was 11.7 ± 17.9 months. The mean left ventricular ejection fraction (LVEF) was 64.3 ± 7.8%, the mean left atrial diameter was 36.4 ± 7.3 mm, and the brain natriuretic peptide (BNP) level was slightly elevated with a mean of 129.5 ± 191.4 pg/ml.

**Table 1:** Patient characteristics

Parameters	
N (male/female)	100 (61/39)
Age (years)	69.3 ± 9.6
Type of AF	
Paroxysmal [n (%)]	68 (68%)
Persistent [n (%)]	20 (20%)
Long-lasting [n (%)]	12 (12%)
Duration of AF (months)	11.7 ± 17.9
Hypertension [n (%)]	49 (49%)
Diabetes [n (%)]	14 (14%)
Structural heart disease [n (%)]	11 (11%)
CHADS2	1.2 ± 1.0
CHA2DS2-VASc	2.3 ± 1.3
LVEF (%)	64.3 ± 7.8
LAD (mm)	36.4 ± 7.3
Ccr (mL/min)	62.0 ± 17.8
BNP (pg/mL)	129.5 ± 191.4

*AF* atrial fibrillation, *LVEF* left ventricular ejection fraction, *LAD* left atrium diameter, *BNP* brain natriuretic peptide

### Details of the ablation procedures performed

The details of the ablation procedures performed are shown in Table 2. Radio-frequency ablations were performed in 82 patients and cryoablations in 18 patients. The mean duration of the procedure was 134.8 ± 38.5 min. As adenosine was not used in five patients with asthma, the total number of patients administered adenosine was 95. The mean dose was 47.9 ± 30.3 mg. In most cases, the basic procedure consisted of inserting electrodes into both PVs and administering a single dose of 30 mg adenosine. However, in cases where individual isolation was performed (e.g., patients who underwent cryoablation), a 30 mg dose was administered to each isolated vessel. The procedure was completed after performing only a PVI in 74 patients. Cavo-tricuspid isthmus (CTI) block lines were created in a total of 14 patients who were clinically found to have typical atrial flutter during ablation or in whom it was induced as a result of ISP. In addition, atrioventricular nodal reentry tachycardia (AVNRT) was induced using ISP in two patients, both of whom had an additional slow-pathway ablation performed. The ablation of non-PV foci was performed in a total of 24 patients.

**Table 2:** Ablation data

Ablation tool	
RF [n (%)]	82 (82%)
Cryo balloon [n (%)]	18 (18%)
Procedure time (min)	134.8 ± 38.5
Adenosine doses (mg)	47.9 ± 30.3
PVI only [n (%)]	74 (74%)
+ CTI block line [n (%)]	14 (14%)
+ SVC isolation [n (%)]	6 (6%)
+ BOX isolation [n (%)]	3 (3%)
+ PMI block line [n (%)]	1(%)
+ Slow-pathway ablation [n (%)]	2 (2%)
+ Non-PV ablation [n (%)]	24 (24%)

*RF* radio-frequency, *PVI* pulmonary vein isolation, *CTI* cavo-tricuspid isthmus, *SVC* superior vena cava, *PMI* peri mitral isthmus

### Dormant conduction

The results obtained using adenosine and ISP are shown in Tables 3 and 4 and those obtained on dormant conduction are shown in Table 3. By using adenosine, dormant conduction was confirmed in 13 patients (13.7%). In these 13 patients, it was temporary in 10 patients and persistent in 3. An additional ablation was performed in persistent cases followed by a second administration of adenosine. No recurrence of dormant conduction was observed in any patients after additional adenosine administration. By using ISP, dormant conduction was observed in 13 patients, and it was persistent in all of them. Therefore, an additional ablation was performed in all 13 cases. There was dormant conduction that was temporarily observed with adenosine and persistently observed with ISP in five cases, and that was temporarily observed only with adenosine and not observed with ISP in five cases. The location of the dormant conduction site observed in a 20-pole ring electrode catheter as a result of adenosine and that observed as a result of ISP administration were the same in all patients. Considering from the results of each additional ablation, the reconnection occurred at one site in all cases. And there were observed in eight cases only with ISP. After an additional ablation was performed for persistent dormant conduction by adenosine, ISP was administered to these patients and no dormant conduction was observed. In 5 patients in whom adenosine could not be used because of asthma, no dormant conduction due to ISP administration confirmed.

**Table 3:** Results after administration of adenosine and isoproterenol. Dormant conduction

Parameters	
Adenosine	
All [n (%)]	13 (13.7%)
Temporary [n (%)]	10 (10.5%)
Persistent [n (%)]	3 (3.2%)
ISP	
All [n (%)]	13 (13%)
Temporary [n (%)]	0 (0%)
Persistent [n (%)]	13 (13%)

*ISP* isoproterenol

**Table 4:** Results after administration of adenosine and isoproterenol. Arrhythmogenic foci

Parameters	
Adenosine	
Arrhythmogenicity of PV	
Automaticity [n (%)]	1 (0.9%)
PV firing-non sustain [n (%)]	2 (2.1%)
PV firing-sustain [n (%)]	0 (0%)
Non-PV foci [n (%)]	3 (3.2%)
ISP	
Arrhythmogenicity of PV	
Automaticity [n (%)]	34 (34%)
PV firing-non sustain [n (%)]	12 (12%)
PV firing-sustain [n (%)]	10 (10%)
Non-PV foci [n (%)]	23 (23%)

*PV* pulmonary vein, *ISP* isoproterenol

**Fig. 2:**
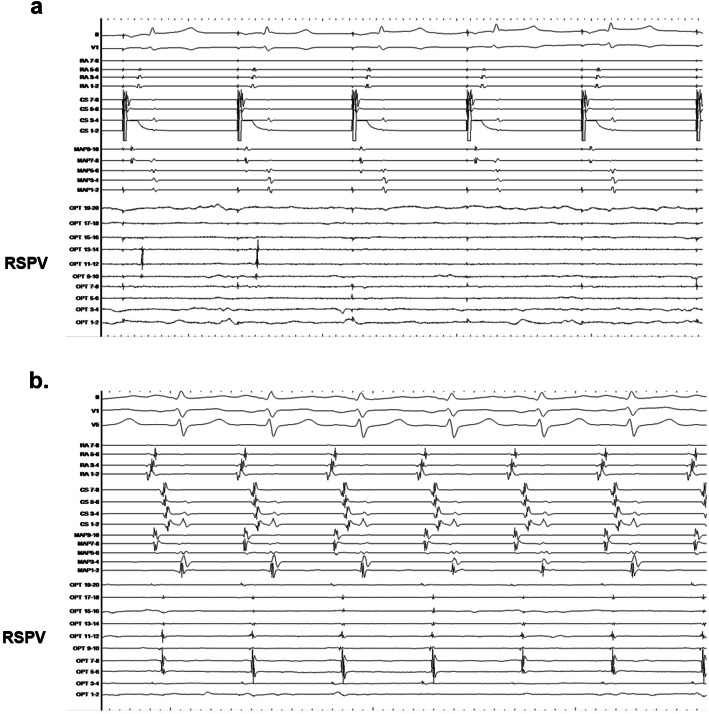
Dormant conduction. **a** An electrocardiogram from a 47-year-old man in whom temporary dormant conduction in the RSPV was observed as a result of administering 30 mg of adenosine after PVI for the treatment of PAF. **b** Since it disappeared after a temporary appearance, the dormant conduction was persistent after the administration of an ISP. RSPV: right superior pulmonary vein; PVI: pulmonary vein isolation; PAF: paroxysmal atrial fibrillation ISP: isoproterenol

An example of a case of dormant conduction is shown in Fig. 2. The patient was a 47-year-old man who was confirmed to have temporary dormant conduction in the right superior PV (RSPV) after the administration of a 30 mg dose of adenosine following PVI in paroxysmal AF (PAF), as shown in Fig. 2a. As the dormant conduction disappeared after a temporary appearance, ISP was administered and the dormant conduction became persistent (Fig. 2b). The ablation was possible during persistent dormant conduction.

### Arrhythmogenic foci

The results of arrhythmogenic foci are shown in Table 4. Only a small number of PVs responded to adenosine. All three patients with non-PV foci were found to have non-PV firing sites. In one patient, SVC firing was observed (SVC isolation was performed in this case), and ablation was impossible in the remaining two cases as the origin was near the sinus node. PV response was observed as a result of ISP administration in over half of the patients. Persistent PV firing was observed in 10 of these patients. This suggests that in these patients, the PVs played a major role in AF. In five patients in whom adenosine could not be used because of asthma, PV automaticity was increased in three cases, non-PV foci also appeared in those three cases, and ablation was performed.

**Fig. 3:**
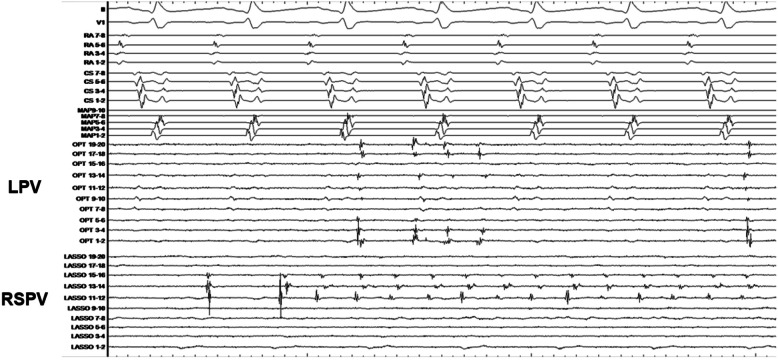
PV firing. An electrocardiogram from a 68-year-old man who was administered with an ISP after a PVI for the treatment of PAF. The LPV was the common branch. Initially, after administration, firing began on both sides and later became persistent in both the PVs. PV: pulmonary vein; ISP: isoproterenol; PVI: pulmonary vein isolation; LPV: left pulmonary vein

A patient with arrhythmogenicity of the pulmonary vein is shown in Fig. 3. The patient was a 68-year-old man who underwent an ISP loading following PVI for PAF. On the left was the common branch. After administration, firing began on both sides. As time elapsed, firing continued in both the PVs. Cases such as this have been assessed to have a high degree of arrhythmia substrate.

The additional ablation of the non-PV foci induced by ISP administration was performed in 23 patients. The details of the 24 patients with non-PV foci, including those in whom SVC firing was induced using adenosine, are shown in Table 5. The location in most of these cases was the SVC.

**Table 5:** Non-PV foci location

N	24
SVC [n (%)]	6 (25%)
RA septum [n (%)]	5 (20.8%)
RA [n (%)]	3 (12.5%)
LA septum [n (%)]	2 (8.3%)
LA posterior [n (%)]	2 (8.3%)
LA anterior [n (%)]	2 (8.3%)
CS [n (%)]	2 (8.3%)
Tricuspid valve [n (%)]	1 (4.2%)
Crista terminalis [n (%)]	1 (4.2%)

*RA* right atrium, *LA* left atrium, *CS* coronary sinus, *SVC* superior vena cava

**Fig. 4:**
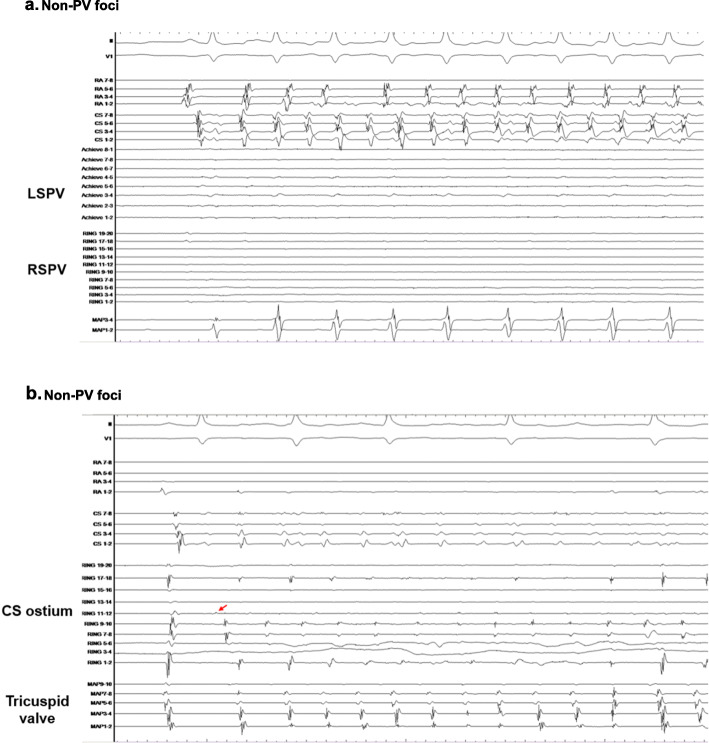
Non-PV foci. **a:** An electrocardiogram from a 68-year-old man after cryoablation of PAF. After ISP administration, the earliest PAC from the ostium of the coronary sinus induced AF. **b:** Multipolar catheter caught the earliest potentials at the CS ostium (red arrow). In this patient, an electric current applied to the posterior wall of the CS ostium caused the PAC to disappear and AF was not induced. PV: pulmonary vein; PAF: paroxysmal atrial fibrillation; ISP: isoproterenol; AF: atrial fibrillation; PAC: premature atrial contraction; CS: coronary sinus

Figure 4 shows a 68-year-old man after cryoablation was performed for PAF.. After ISP administration, the earliest PAC from the ostium of the coronary sinus induced AF (Fig. 4a). The precise location of the non-PV origin was identified by mapping and defibrillation (Fig. 4b). In the case of this patient, ablation performed at the posterior wall of the CS ostium led to the disappearance of the PAC, which prevented the induction of AF.

## Discussion

### Significance of this study

AF occurs in conjunction with supraventricular extrasystoles. Many premature contractions originate in the PV, and ablation targeting the premature contractions leads to the disappearance of AF [5]. Many cases of AF originate in the PV; thus, the left PV atrial junction and intra-PV re-entry play important roles in the persistence of AF [6]. In all types of AF, PVI is performed first. In cases of non-PV foci, additional ablation is performed on each relevant trigger. Non-PV foci have been reported in approximately 10–20% of patients [7–10]. The outcomes after ablation therapy in patients with non-PV foci are poor [10, 11], but the outcomes in cases where ablation is possible are better than in those where it is not possible [12]. Therefore, regardless of whether the AF is paroxysmal or persistent, in cases of non-PV foci, ablation should be attempted. The common sites of non-PV foci include the SVC, left atrium posterior wall, crista terminalis, CS, and vein or ligament of Marshall. The atrial muscle extends anywhere from several millimeters to several centimeters into the veins that are the main origins of the non-PV foci. It is thought that due to the presence of sino-atrial node cells and cells similar to Purkinje fibers and abnormal automaticity (triggering activity), ectopic excitation is liable to occur [13–15].

The non-recurrence rate of paroxysmal AF in cases after PVI is around 80% [16–18] and that of persistent AF is approximately 60% [19]. Postoperative recurrence is a serious problem that is caused due to reconduction of the isolated PVs and the onset of non-PV foci.

Adenosine is used to confirm the presence of post-PVI reconduction. Adenosine administration shows PV dormant conduction; and thus, it allows for the identification of PVs that are at risk of reconduction [20, 21]. However, in many cases dormant conduction is temporary, it makes it difficult to ablate and the clinical effectiveness of additional ablation in such cases remains a point of debate [22].

In the present study, our objectives were to elucidate post-PVI dormant conduction, confirm PV arrhythmia substrates, and induce non-PV foci. This was done by administering a high dose of ISP in addition to adenosine and then observing their effects during the acute stage.

### Dormant conduction

The shortening of the action potential duration and the refractory period by ISP administration is considered to be the factors that cause dormant conduction [23]. As most cases of dormant conduction caused by adenosine were temporary, it was necessary to administer adenosine repeatedly. The administration of ISP resulted in persistent conduction that made the ablation procedure possible. It is considered that the half-life of ISP is longer than that of adenosine, which is the main factor that caused persistent dormant conduction. The location of the dormant conduction observed in a 20-pole ring electrode catheter caused by adenosine and ISP was the same in all patients, and no effect other than persistent dormant conduction was observed. However, when one or the other drug was administered alone, the dormant conduction was not induced in some patients. It is reported that to identify and target the dormant PV conduction during catheter ablation of atrial fibrillation is a highly effective strategy to improve arrhythmia-free survival [20], and hence, the use of ISP is useful for AF ablation.

### Arrhythmogenic foci

Some reports have confirmed the effects of adenosine and ISP on the search for arrhythmogenic foci. It is reported that adenosine induces AF triggers especially from RA and SVC, but the clinical significance of these foci is questionable [24, 25]. On the other hand, ISP-induced AF is more likely initiated from the PVs as compared to that after the administration of adenosine [24]. We also found that all adenosine-induced AF was from RA and SVC. Furthermore, we were the first to evaluate the arrhythmic substrate in PVs after PVI. In patients in whom clear arrhythmia substrates were observed only in the PV, only a PVI was performed, and we found that this was a useful strategy. This is particularly significant in cases of persistent AF. Even in the cases of non-PV foci, as frequent occurrences were observed not only in cases of firing but also in those in whom PAC occurred, it was easy to identify the origin and we were able to confirm that performing an ablation was successful.

### Efficacy of ISP

In this study, we confirmed the acute effect of ISP in patients who underwent AF ablation. ISP acts on the beta-receptors of the sympathetic nerves, thereby increasing the heart rate and promoting conduction. As a result, the automaticity of the heart is promoted, resulting in a high degree of excitation of arrhythmia substrates and the occurrence of premature contractions. Thus, attempts have been made to utilize these effects during catheter ablation. A dose of 6 μg/min was administered for 5 min, and this was uniform across all patients in the study. Although the administration caused an elevation in heart rate and a decrease in blood pressure, none of the patients experienced any effects on their circulatory dynamics or adverse effects at the dose utilized in our study. The effects of administering ISP disappeared approximately 15 min after administration was completed, useful observations were made in many patients during this period and no major postoperative problems were observed.

There are two significant results we would like to emphasize in this study. First, the temporary dormant conduction confirmed by adenosine was all sustained by the administration of ISP. Secondly, we were able to confirm the arrhythmic substrate of PVs after PVI. Based on the result of this study, we believe that ISP plays an extremely important role in cases of atrial fibrillation ablation. But it should be noted that the effects of ISP are not inclusive of all the effects of adenosine. There were cases where useful findings were observed in patients who were administered with adenosine only. Therefore, ISP should not be considered as a substitute for adenosine but as a complement to it.

### Study limitations

This study had several limitations. First, the number of patients was small because it was a prospective study conducted at a single institution. Regarding this, the accumulation of cases is required in the future. Second, the order of administration of adenosine and ISP may be a limitation. The half-life of adenosine was shorter than that of ISP, and adenosine was used first. Therefore, the order of administration may have affected the results of our study. Third, although the positions of dormant conduction observed in the multipolar catheter were the same by adenosine and ISP administration, it could not be said that they coincided with each other. Lastly, in this study, the effectiveness was confirmed in the acute stage. However, an identification of the long-term prognosis will require further study.

## Conclusions

In this study, ISP was used following a PVI in order to identify dormant conduction and arrhythmogenic foci. The results showed that ISP was effective in the acute stage. The dormant conduction that occurred as a result of ISP administration was persistent, which is useful when ablation is performed. Our results also suggest that it is useful in confirming PV arrhythmia substrates and inducing non-PV foci. However, these effects may have been a result of adjunct therapy of ISP with adenosine; and therefore, we believe that the combined use of the two drugs is a useful approach. Further studies evaluating the long-term prognosis of this intervention in AF ablation must be performed.

## Data Availability

The datasets used and analysed during the current study are available from the corresponding author on reasonable request.

## Abbreviations

AF: Atrial fibrillation
BNP: Brain natriuretic peptide
CS: Coronary sinus
CTI: Cavo-tricuspid isthmus
LA: Left atrium
LAD: Left atrium diameter
LVEF: Left ventricular ejection fraction
PAC: Premature atrial contraction
PAF: Paroxysmal atrial fibrillation
PMI: Peri mitral isthmus
PV: Pulmonary vein
PVI: Pulmonary vein isolation
RA: Right atrium
RF: Radio-frequency
RSPV: Right superior pulmonary vein
SVC: Superior vena cava
